# Immunohistological responses in mice implanted with Parylene HT – ITO ECoG devices

**DOI:** 10.3389/fnins.2023.1209913

**Published:** 2023-08-31

**Authors:** Miklós Madarász, Flóra Z. Fedor, Zoltán Fekete, Balázs Rózsa

**Affiliations:** ^1^BrainVision Center, Budapest, Hungary; ^2^János Szentágothai PhD Program of Semmelweis University, Budapest, Hungary; ^3^Laboratory of 3D Functional Network and Dendritic Imaging, Institute of Experimental Medicine, Budapest, Hungary; ^4^Research Group for Implantable Microsystems, Faculty of Information Technology and Bionics, Pázmány Péter Catholic University, Budapest, Hungary; ^5^Sleep Oscillation Research Group, Institute of Cognitive Neuroscience and Psychology, Research Center for Natural Sciences, Budapest, Hungary; ^6^Two-Photon Measurement Technology Research Group, The Faculty of Information Technology, Pázmány Péter Catholic University, Budapest, Hungary; ^7^Femtonics Ltd., Budapest, Hungary

**Keywords:** microECoG, parylene, polymer implants, immunohostochemistry, foreign body response

## Abstract

Transparent epidural devices that facilitate the concurrent use of electrophysiology and neuroimaging are arising tools for neuroscience. Testing the biocompatibility and evoked immune response of novel implantable devices is essential to lay down the fundamentals of their extensive application. Here we present an immunohistochemical evaluation of a Parylene HT/indium-tin oxide (ITO) based electrocorticography (ECoG) device, and provide long-term biocompatibility data at three chronic implantation lengths. We implanted Parylene HT/ITO ECoG devices epidurally in 5 mice and evaluated the evoked astroglial response, neuronal density and cortical thickness. We found increased astroglial response in the superficial cortical layers of all mice compared to contralateral unimplanted controls. This difference was largest at the first time point and decreased over time. Neuronal density was lower on the implanted side only at the last time point, while cortical thickness was smaller in the first and second time points, but not at the last. In this study, we present data that confirms the feasibility and chronic use of Parylene HT/ITO ECoG devices.

## Introduction

Today, polymers are playing a dominant role in the design and development of implantable microdevices (Fekete and Pongrácz, 2017). The era of microscale devices that can be inserted into the tissue has been revolutionized by substrate materials like Polyimide (Zátonyi et al., 2018), PDMS (Miranda et al., 2021), Parylene C (Golda-Cepa et al., 2020), shape memory polymers (Fedor et al., 2022), hydrogels (Goding et al., 2019) and PET (Kunori and Takashima, 2015). Among them, poly[chloro-p-xylylene]or shortly Parylene C (trade name) is one of the most popular structural materials of biomedical devices (Meng et al., 2008; Castagnola et al., 2015; De La Oliva et al., 2018). The advantage of the thin layer of thermoplastic Parylene is that it can be formed by chemical vapor deposition at low ambient temperature. The deposited layer is conformal, pin-hole free, chemically inert, while showing low vapor permeability (Tan and Craighead, 2010). Derivatives of Parylene differ from one another in functional groups replacing hydrogen atoms on the phenyl ring or on the aliphatic bridge. In the case of Parylene C, the dimer is replaced by a chlorine atom. Besides the extensive use of Parylene C, another derivative Parylene HT (or poly[α,α,α′,α′-tetrafluoro-p-xylylene]) has gained attention. For Parylene HT, a fluorine atom replaces the alpha hydrogen on the aromatic ring. This slight change in its chemical structure, however, leads to several advantageous properties. Just like Parylene C, its fluorinated variant, HT also meets the ISO 10993 biocompatibility standards (Kumar, 2010). On the other hand, Parylene HT shows better performance in many applications due to its lower dielectric constant (Diaham et al., 2014), lower moisture absorption and stability in high-temperature processes – long-term stability at 350°C and under UV exposure (Lu et al., 2010). The beneficial dielectric properties have been leveraged in the fabrication of electrowetting platforms (Dhindsa et al., 2011). In the case of certain conductive materials with high melting point, e.g., iridium, patterning on Parylene HT is still compatible with the potentially high deposition temperature in contrast to Parylene C (Rodger et al., 2008). Regarding biomedical applications, there are only a few demonstrations that exploited the features of this material either as coating or substrate. The work of Rodger et al. pointed out for the first time that Parylene HT can be used as a substrate of neural microelectrodes designed for charge delivery into the tissue (Rodger et al., 2008). Rios et al. has utilized Parylene HT coating on a 3D multi-shank neural probe (Rios et al., 2016). Shang et al. has recently demonstrated that an oxygen-transporting mesh composed of Parylene HT is able to improve cell viability in cell transplantation processes (Shang et al., 2021). Mechanical and optical properties of Parylene HT were jointly exploited when cell culturing membranes with imaging options were prepared and successfully tested (Kim et al., 2014). The tunable gas permeability of Parylene HT was confirmed by Scianmarello and colleagues, who reported on its application for culturing HEK-293 cells (Scianmarello et al., 2019). Using hNT astrocyte cells, a study comparing various derivatives of Parylene as cell attractive substrates concluded that the quality of cell patterning on the HT variant is the same as on Parylene C, making it a similarly suitable substrate for cell patterning (Raos et al., 2019). The optical properties of Parylene HT are also superior to Parylene C, especially the remarkably lower auto-fluorescence of the material, which is highly beneficial for multimodal neuroimaging schemes, e.g., combining fluorescent microscopy with high-density electrophysiology. More recently, our group has demonstrated that by combining Parylene HT with indium tin oxide, a fully transparent cortical microelectrode array can be fabricated. The optical and electrochemical analysis of this device, as well as simultaneous electrophysiology and fluorescence imaging of awake mice for up to 51 days was presented by Zátonyi et al. (2020). Chronically implanted neural interfaces need to function over extended time periods. One of the main reasons for unreliable electrical recordings in chronic experiments is the prolonged presence of ECoG devices resulting in neuroinflammatory responses that evoke glial scar formation, eventually isolating the device from the tissue (Bérces et al., 2016; Kim et al., 2019). Therefore, the importance of investigating the performance of different neural interface materials can not be understated, a fact well recognized by the field (Vomero et al., 2020; Fedor et al., 2022; Fekete et al., 2023). Several methods are available to probe the effects of device implantation. Immunohistological staining of brain slices for Glial Fibrillary Acidic Protein (GFAP) labels astroglia, a major contributor to glial scars. NeuroTrace, a fluorescent Nissl staining, is a suitable indicator for neuron damage and ongoing foreign body response, as the substance redistributes within the cell body in injured or regenerating neurons. Comparison of cortical thickness may be indicative of neuronal death or a depression of the cortex due to low conformability of the implanted device (Eng, 1985; Quinn et al., 1995; Hanz and Fainzilber, 2006; Fedor et al., 2022). Although the successful use of Parylene HT *in vitro* and *in vivo* has been demonstrated earlier, the chronic response of the tissue or cell population to this material is still required. To investigate these questions, we performed immunohistochemical staining of brain samples implanted with a previously reported Parylene HT / ITO device (Zátonyi et al., 2020).

## Methods

Parylene HT, a coating material for electronic components, gains more and more interest, although its effect on the cortex has never been evaluated with immunohistochemical methods. Here, we analyzed data of DAPI, NeuroTrace and GFAP stained slices, comparing fluorescence intensity of stainings, neuron density and cortical thickness of implanted areas with contralateral control regions. Regions of interests denoted in the superficial (I-IV) and deep (V-VI) layers were differentiated, as a difference between environments close or remote to the electrode was hypothesized to be affected differently (Fedor et al., 2022). DAPI staining was used to stain cell nuclei to follow up the overall effect of the electrode on every cell and as a background for cortical thickness measurements. NeuroTrace staining was chosen for calculating neuronal density and as an indicator of incidental neuronal damage together with cortical thickness. GFAP staining was used to label astrocytes, which are an important part of the immunological response. To observe the time dependence of the effects resulting from different implantation lengths, 1–1 mice with shorter and longer implantation lengths were also examined.

### Device

**Figure 1:**
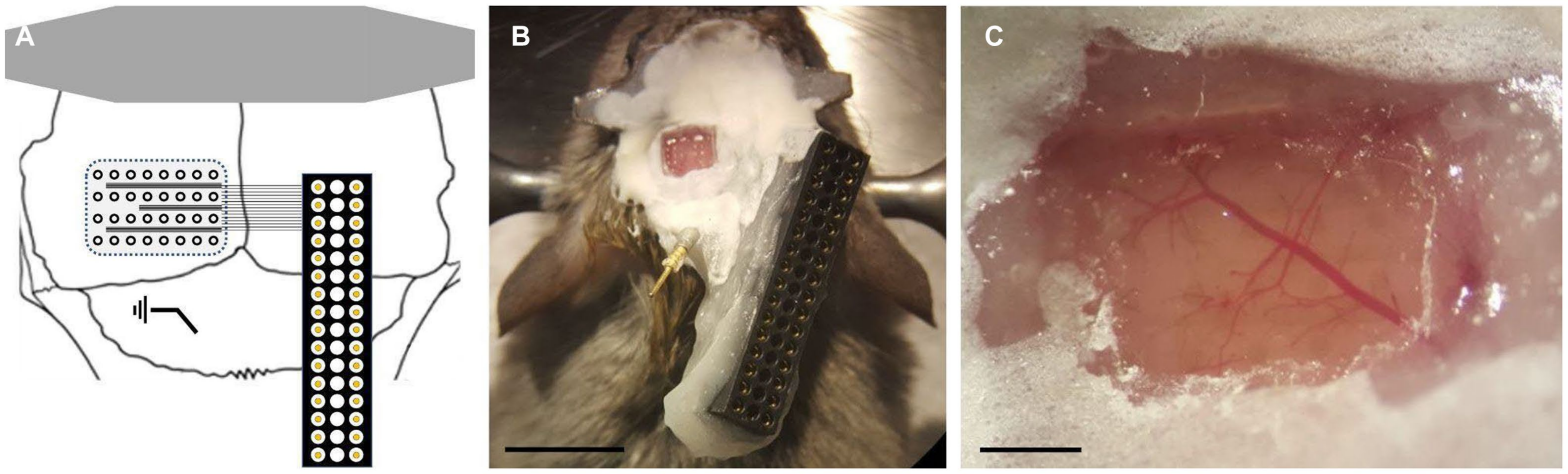
Parylene HT device and implantation. **(A)** Schematics of the surgical arrangement (not to scale). **(B)** Localization of the electrode array in actual surgical footage. Scale bar shows 5 mm. **(C)** Cleaned surgical area for the electrode above the dura mater relative to vascular network. Scale bar shows 500 μm.

The 32-channel microECoG device holds recording sites of 150 μm in diameter with 500 μm as inter-site distance. The outline of the sensor arrays is 4.5 mm × 5.5 mm, which fully covers the exposed brain surface. The device is composed of a 100 nm thick indium tin oxide conductive film embedded into a 16 μm thick double-layer of Parylene HT. Conductive traces and bonding pads are also made from indium tin oxide, which were connected to a PreciDiP connector (see Figure 1A for schematic view). To create the microECoG, standard MEMS technology processes were used. The whole fabrication scheme, including data on specific process parameters, are detailed in our previous paper (Zátonyi et al., 2020).

### Animals

All procedures were in accordance with the Hungarian Act of Animal Care and Experimentation [2013, (II.14), section 40/2013] and approved by the Animal Care and Use Committee of the Government Office (approval: PE/EA/674–4/2019). Five male FVB/Ant mice expressing GCaMP6f under the thy1 promoter, aged 117–149 days, were implanted with transparent micro ECoG devices. After an implantation period of 42–116 days (Table 1), the mice were sacrificed and subjected to immunohistochemical analysis. The animals were housed under controlled temperature, humidity, and lighting, with *ad libitum* access to food and water.

**Table 1:** Experimental animals.

Animal	Implantation length	Age at implantation	Age at perfusion	Number of evaluated slices
M1	42	143	185	18
M2	75	149	224	18
M3	76	148	224	16
M4	83	145	228	17
M5	116	117	233	18

Three groups were formed for analysis according to the duration of implantation (M1, M2–4, M5), marked with shades of blue. Implantation length and age are given as days.

### Device implantation

The process of device implantation was identical to the one described in the previous work of our group (Zátonyi et al., 2018). Briefly, the mice were anesthetized with fentanyl (0.05 mg/kg), medetomidine (0.5 mg/kg) and midazolam (5 mg/kg). Ropivacaine (50 μL) was also administered for local analgesia. The top of the scalp was removed, and a rectangular craniotomy (3-by-4 mm) was drilled over the left hemisphere with a 0.3 mm drill bit, fit to the corner defined by the midline and the lambda sutures. The device was placed on the brain surface and covered with a rectangular cover glass fitting into the window and a larger one covering the edges of the craniotomy. The cover glasses were pushed down to ensure the tight adherence of the device to the dura and sealed using a mix of dental cement (Relyx) and cyanoacrylate glue. A reference electrode was placed above the cerebellum. A thin steel plate was bonded to the skull over the right hemisphere to provide stability for the electrical connector, which was then bonded to it with the same glue mix as used before. Finally, a steel headbar was fixed to the skull with dental cement (Superbond) (Figure 1). Animals were awakened with revertor (2.5 mg/kg), flumazenil (2.5 mg/kg) and nexodal (1.2 mg/kg) and left to rest for at least 3 days.

### Tissue preparation and immunofluorescent staining

Following implantation, mice were deeply anesthetized with isoflurane (Isoflurane, 0.5–1%, Forane, Abbott) and transcardially perfused with 4% (w/v) formaldehyde. After 20 min of perfusion the brain was removed after detaching the electrode grid from the cortical surface. Brains were postfixed (4% formaldehyde) until immunofluorescent staining was performed. Brain blocks were cut into coronal sections (40 μm, cryo) on a sliding microtome (Leica SM2010 R) and stored in 24-well plates in 1% PBS with 0.3% Sodium Azide. Free-floating sections were washed 3 times for 10 min with 1X PBS, then blocked for an hour at room temperature in a blocking buffer (5% Bovine Serum Albumin and Goat Serum (Thermo Scientific) containing 0.5% Triton-X (Sigma) for membrane permeabilization). After blocking and following 3 subsequent 10-min washing steps, first with 0.3% Triton-X then with 1% PBS, GFAP primary antibody (1:2500, chicken anti-GFAP, #PA1-10004, Invitrogen) was applied directly. Slices were kept at 4°C overnight before additional 3 subsequent 10-min washing steps and the application of the species-specific secondary antibody (1:200, goat anti-chicken Alexa Fluor 647, #A-21449, Invitrogen). In addition to the secondary antibody, green fluorescent Nissl stain (1:400, NeuroTrace, #N21480, Invitrogen) was applied to the tissue sections for 2 h at room temperature. Unbound secondary antibodies were then washed off with 1X PBS (3×10 min). Sections were mounted using Fluoromount-G mounting medium containing DAPI (#00–4,959-52, Invitrogen) to co-label total cell nuclei. The slides were stored at 4°C in darkness until immunofluorescent imaging was performed the next day.

### Image analysis

**Figure 2:**
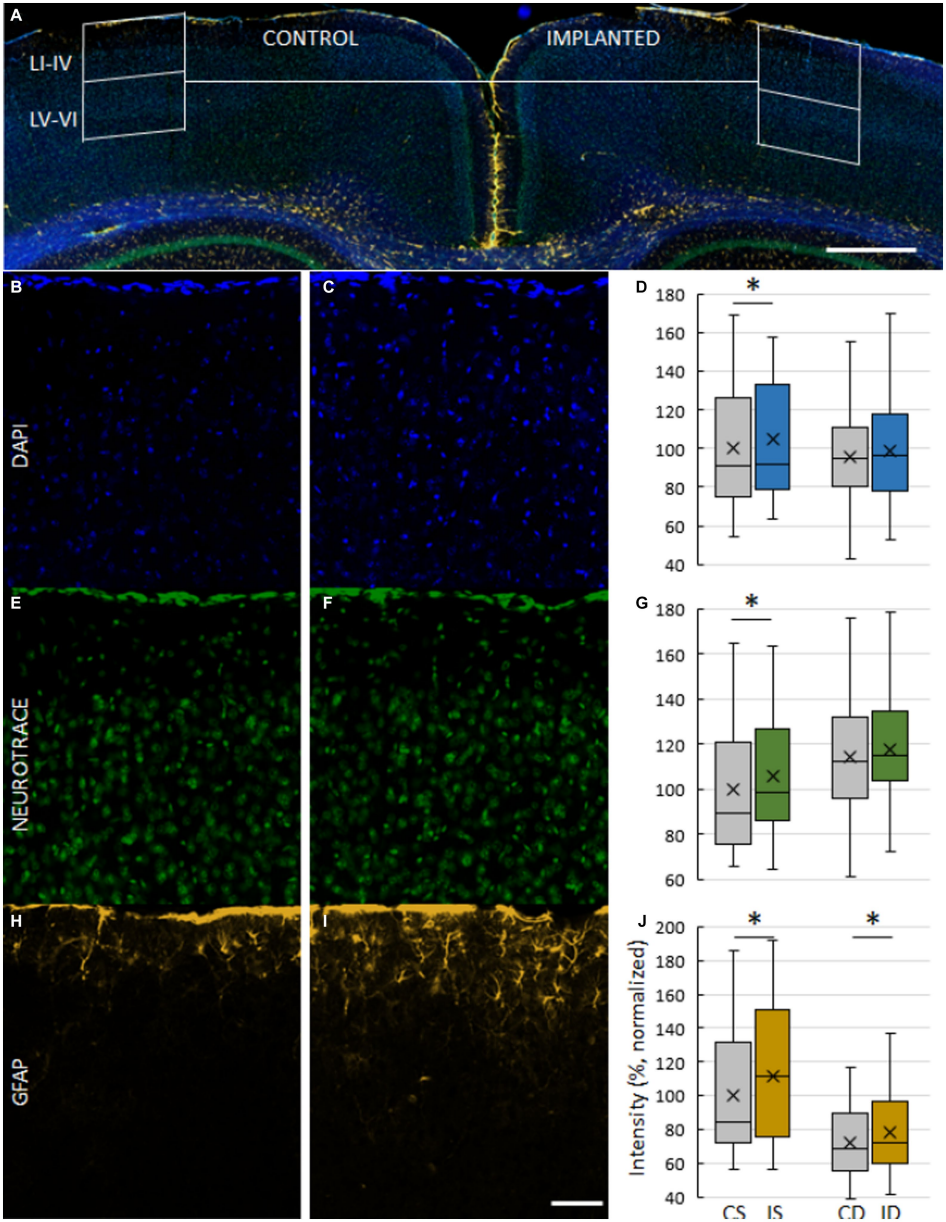
Images of a Parylene HT/ITO ECoG implanted brain after 75 days of chronic implantation (M2). **(A)** Composite image of a coronal slice showing the arrangement of ROIs on control **(B,E,H)** and implanted **(C,F,I)** cortex. ROIs are divided into superficial (layers I–IV) and deep (layers V–VI). Magnification 3.2X, scale bar 500 μm. **(B,C)** Cell nuclei stained with DAPI. **(E,F)** Neurons stained with NeuroTrace, a fluorescent Nissl stain. **(H,I)** Astrocytes labeled with GFAP. Magnification 20X, scale bar 50 μm. **(D,G,J)** Comparison of ROI fluorescent intensity of DAPI **(D)**, NeuroTrace **(G)** and GFAP **(J)** labeling. Mean ROI intensity was normalized to ROI area and presented here as a percentage of the mean of control, superficial ROIs of their respective stainings. CS – control, superficial. IS – implanted, superficial. CD – control, deep. ID – implanted, deep. Asterisks denote significant differences (*p* < 0.05).

Fluorescent images were acquired using a Slide Scanner (Pannoramic MIDI II, 3DHISTECH) equipped with a 20X objective under blue (DAPI filter), green (FITC filter) and far red (Cy5 filter) illumination. The digital images were processed in CaseViewer (3DHISTECH) and were exported at 300 dpi in TIFF file format. Animals were arranged into three groups for analysis according to the duration of implantation (M1, M2-4, M5) (Table 1). Histological slices that were determined to originate from the implantation area by comparison of surgical coordinates and the slices’ position from bregma according to The Mouse Brain atlas were included in the analysis (Franklin and Paxinos, 2001). This resulted in 16–18 contiguous slices from each animal (Table 1). Regions of interest (ROI) were then manually drawn on cortical areas that were under the implanted device and mirrored contralateral areas. Superficial (layers I-IV) and deep (layers V-VI) layers were distinguished with separate ROIs for separate analysis of upper and lower layers. Image analysis was performed in MATLAB (Mathworks). Raw fluorescence intensity values of every pixel in a ROI were averaged and then normalized to the ROI area (mean intensity / mm^2^). Neuronal density was calculated using the ITCN plugin of ImageJ (NIH). Neurons inside ROIs were counted on images of NeuroTrace stained slices. Counted cells in superficial and deep ROIs were summed and normalized to the summed area of both ROIs (cells / μm^2^) for comparison between implanted and control sides. For presentation purposes, values were expressed as a percentage of the mean of control, superficial ROIs on Figure 2; Supplementary Figures S1, S2, which is indicated in the figure legends. Cortical thickness was measured in CaseViewer (3DHISTECH). Lines matching the inclination of the brain surface at the position of the ROIs were drawn on the slices, and cortical thickness was measured perpendicular to these lines with the distance measurement tool of CaseViewer.

### Statistics

Statistical evaluation was performed in Prism 6.0 (GraphPad). Data groups selected for comparison were tested to be normally distributed with the D’Agostino & Pearson test, Anderson-Darling test, Shapiro–Wilk test and Kolmogorov–Smirnov test. If any of these four tests reported the groups to be normally distributed, a paired t-test was used, otherwise, a Wilcoxon matched-pairs signed rank test was used. Every test was two-tailed with Type I error rate of 0.05. Data is presented as mean ± standard deviation. Whiskers of box plots show minimum and maximum values within Q1–1.5 × IQR and Q3 + 1.5 × IQR, respectively. Data points outside of this range are considered to be outliers.

## Results

**Figure 3:**
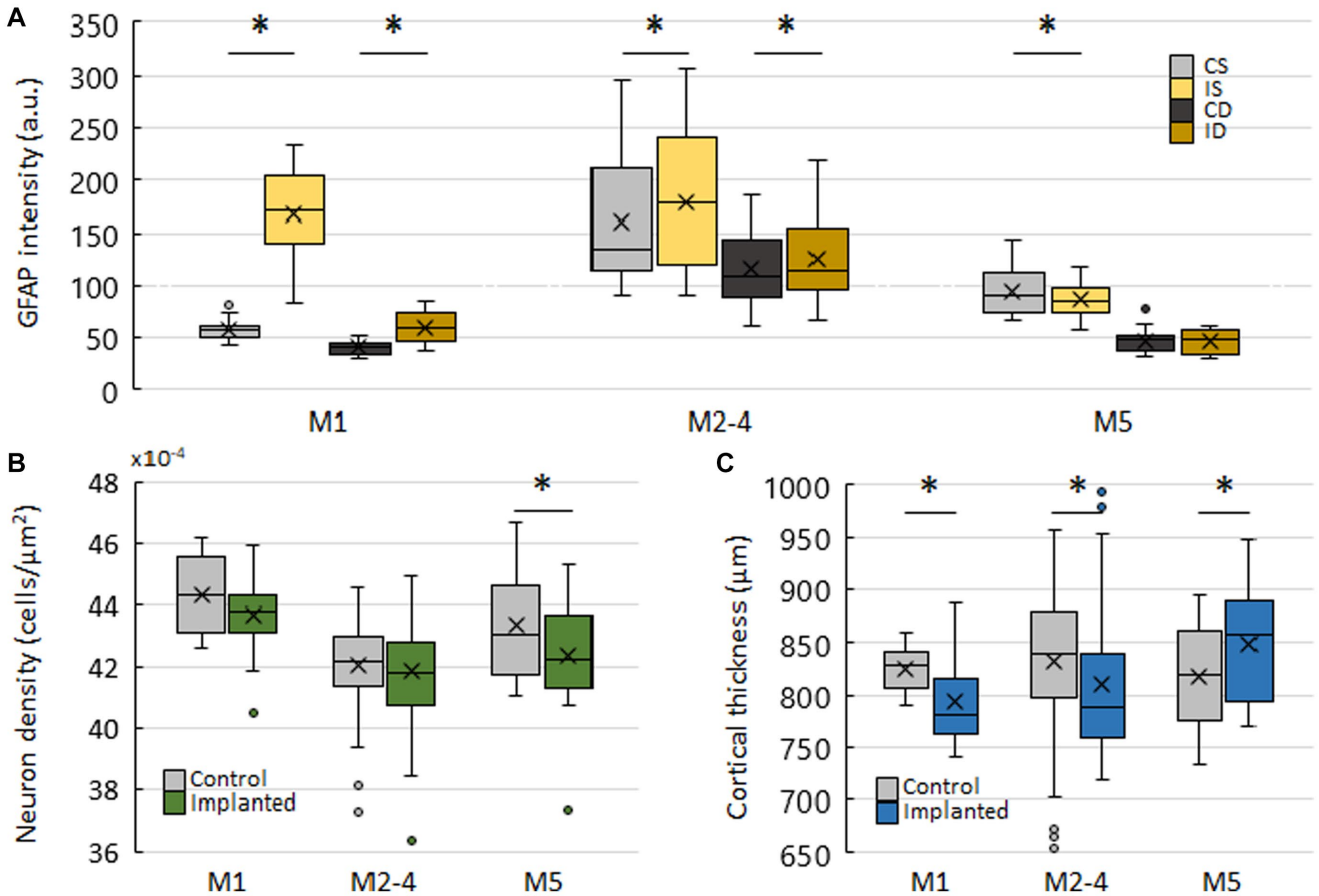
Characterisation of implanted and control hemispheres in three implantation lengths. **(A)** Comparison of fluorescence intensities of GFAP stainings between superficial and deep ROIs on control and implanted hemispheres. Mean ROI intensity was normalized to ROI area. CS – control, superficial. IS – implanted, superficial. CD – control, deep. ID – implanted, deep. **(B)** Neuron density on control and implanted hemispheres. Counted cells in superficial and deep ROIs were summed and normalized to the summed area of both ROIs. **(C)** Cortical thickness on control and implanted hemispheres. Asterisks denote significant differences (*p* < 0.05).

Examination of M2-4, implanted for 75, 76 and 83 days, served as the starting point of the evaluation (Figure 2). Intensity of DAPI and NeuroTrace stainings were higher in superficial ROIs of M2-4 on the implanted side (DAPI: 4.96% ± 28.94%, *p* = 0.0123; NeuroTrace: 5.41% ± 24.73%, *p* = 0.0165), but not in deep ROIs (Figures 2A–G). A higher NeuroTrace intensity is a sign of elevated protein synthesis capacity, indicative of Nissl substance redistribution to the periphery of cells in injured or regenerating neurons. Intensity of GFAP stainings were moderately higher on both superficial (11.21% ± 38.51%, *p* = 0.0018) and deep ROIs of M2-4 (7.56% ± 33.94%, *p* = 0.0042) (Figures 2H–J, 3A), indicating an elevated astroglial presence after a considerably long period of implantation. While neuronal density was not different between the implanted and control side (Figure 3B), the cortex on the implanted side was thinner (−23.36 μm ± 66.34 μm, *p* = 0.0201; Figure 3C).

In M1, implanted for 42 days, the intensity of DAPI and NeuroTrace stainings were higher in superficial ROIs (DAPI: 12.07% ± 18.15%, *p* = 0.0142; NeuroTrace: 8.27% ± 17.63%, *p* = 0.0482), but not in deep ROIs (Supplementary Figures S1A–G), just as in M2-4. The intensity of GFAP staining was greatly increased in both superficial (187.78% ± 71.61%, *p* < 0.0001) and deep ROIs (50.18% ± 38.45%, *p* = 0.0361) (Supplementary Figures S1H–J; Figure 3A), indicating the ongoing peak astroglial response. Although the difference in neuronal density was nonsignificant (Figure 3B), the cortex on the implanted side was again thinner (−32.49 μm ± 42.13 μm, *p* = 0.0045) (Figure 3C). Generally, results of M1 mirror those of M2-4 with a larger magnitude, suggesting an attenuation of the observed effects over time (Figure 3A). Moreover, the significant differences in labeling intensities of M1 and M2-4 on superficial, but not on deep ROIs suggests a greater and more localized impact of implantation on superficial layers.

In M5, implanted for 116 days, the intensity of DAPI and NeuroTrace stainings were significantly lower in the superficial ROIs on the implanted (DAPI: −10.53% ± 26.44%, *p* = 0.0083; NeuroTrace: −16.2% ± 13.55%, *p* = 0.0003), but not in deep ROIs (Supplementary Figures S2A–G). The intensity of GFAP staining was also lower in superficial ROIs of the implanted side (−8.13% ± 17.74%, *p* = 0.0361) (Supplementary Figures S2H–J; Figure 3A). Contrary to M1 and M2-4, neuronal density of the implanted side in M5 was significantly lower (−2.32% ± 4.13%, *p* = 0.015) (Figure 3B). This was paired, however, with a significantly thicker cortex (31.26 μm ± 33.21 μm, *p* = 0.0009) (Figure 3C). The lower intensity of the three stainings on the implanted side in M5 is noteworthy. In the sense of continuity, it complements the attenuating trend of M1 and M2-4, although a balanced or slightly higher intensity on the implanted side was more expected. The thicker cortex on the implanted side also corresponded to the intensity data, while neural density deviated from the tendency of the results of M5. Finally, as in M1 and M2-4, the differences between control and implanted ROIs were larger in the superficial ROIs.

## Discussion

The Parylene HT / ITO ECoG device presented here was developed for recording of neuronal signals and fluorescent imaging simultaneously and chronically over months of implantation. The abilities of this electrode has been demonstrated previously with electrophysiological measurements and two - photon imaging, concluding that the Parylene HT / ITO ECoG electrode is “a good candidate to provide large area and high-resolution cortical mapping of electrophysiology and simultaneous optical observation of neuronal ensembles on a cellular level” (Zátonyi et al., 2020). Over extended periods of chronic implantation, it is necessary to evaluate the immune response to the implantation of a device and the presence of its material components, yet an immunohistochemical evaluation of the substrate has not been presented to date. Here, we evaluated the immune response to the Parylene HT ECoG device implantation 42 to 116 days post implantation with immunohistochemical staining of DAPI, NeuroTrace and GFAP, comparison of neuronal density and cortical thickness.

### Time dependence of labeling intensity

GFAP labeling, indicative of the astroglial response, recapitulated the described response to cranial surgery (Holtmaat et al., 2009; Koletar et al., 2019). We found a major increase in its intensity at the shortest time point relative to the control side. This difference between control and implanted side appeared to be more localized to the superficial layers, as the difference was smaller in deep ROIs. DAPI and NeuroTrace intensity supported this observation, as differences in these labelings were only observed in superficial layers. This is in good agreement with the formation of glial scars in the immediate vicinity of implanted devices and shows the higher exposure of superficial layers to surgery induced changes. We also observed that the difference between control and implanted sides decreased between the first and middle time points, and reversed in the last in all three stainings. Together with the relative preservation of deeper layers, this is promising for imaging of deeper cortical layers long after implantation, and may advocate the adaptation of longer implantation periods in chronic imaging efforts. When comparing absolute levels of GFAP intensity between different time points, we found that control and implanted ROIs of M2-4, particularly superficial ROIs, were comparable to the highly elevated superficial ROIs of M1 (Figure 3A). The GFAP levels of superficial ROIs in M5 also display a similar profile. While the statistical testing of this is hindered by the overall number of animals, the causes of this apparent elevation may be speculated. A possible explanation may be an increase in baseline GFAP levels at this implantation time, as the differences between control and implanted sides are preserved.

Changes in cortical thickness or neuronal density may be indicative of an ongoing or concluded immune response and are therefore useful in evaluating the impact caused by the implantation of the device. Although it could be expected that the total width of the cortex would correlate with the neuronal density, the results showed a different picture. Despite unchanged neuronal density in the first two time points, the thickness of the cortex was reduced significantly in M1 and M2-4 (Figure 3C), while neuron density was lower only in M5, where cortical thickness was actually larger on the implanted side.

The evoked immune response encountered here, particularly GFAP and NeuroTrace labeling, is in line with previous reports of immune response after cranial window (Holtmaat et al., 2009) or epidural device implantation (Fedor et al., 2022). GFAP expression increases 1–2 weeks after surgery ipsilaterally and remains elevated, to a lesser degree, for up to 6 months (Brosch et al., 2020), although there are reports of regained baseline levels (Koletar et al., 2019; Park et al., 2019). The magnitude of these increases covers a wide range, from 17% (Brosch et al., 2020; Fedor et al., 2022) and 50% (Holtmaat et al., 2009) to 300% (Schweigmann et al., 2021). In some cases, the reported increase is not quantified (Xu et al., 2007; Guo et al., 2017; Brodnick et al., 2019; Koletar et al., 2019), or the amount of increase is not reported (Park et al., 2019). Importantly, this agreement with previous reports shows that the implantation of Parylene HT did not evoke an immune response that would exceed the responses evoked by a common craniotomy window surgery.

Softness and flexibility is hypothesized to be an important contributor to the biocompatibility of a device and its effect can be quantified by measuring the depression of the cortex (or cortical thickness) caused by a device (Vomero et al., 2020). Here, we found that implanted cortices were slightly thinner in the first two implantation lengths, by 32 and 23 μm on average respectively, and slightly thicker at the last time point, by 31 μm on average. While the flexibility of Parylene HT is certainly surpassed by shape memory polymers (Fedor et al., 2022) and hydrogel - based devices, its impact here manifests in tens of micrometers on average.

It is important to note that the data presented here may be limited by the overall number of implanted mice. In particular, the shortest (M1) and longest (M5) implantation lengths had only one mouse examined. While the initial GFAP response after craniotomy, corresponding to M1, is well described, further testing would certainly strengthen the results of the longest implantation period. Nevertheless, our results correspond well to the described effects of cranial implantation surgery (Holtmaat et al., 2009; Schweigmann et al., 2021) and do not suggest an additional, aggravating element introduced by the materials of the ECoG device. Therefore, we recommend Parylene HT as a stable and biocompatible substrate for chronically implanted transparent neural interfaces.

## Data availability statement

The original contributions presented in the study are included in the article/Supplementary material, further inquiries can be directed to the corresponding authors.

## Ethics statement

The animal studies were approved by Animal Ethics Committee of the Institute for Experimental Medicine. The studies were conducted in accordance with the local legislation and institutional requirements. Written informed consent was obtained from the owners for the participation of their animals in this study.

## Author contributions

MM performed the *in vivo* experiments. FF performed the staining and the image processing. MM, FF, and ZF performed the analysis and evaluated the results. MM, FF, ZF, and BR wrote the manuscript. ZF and BR provided funding of the experiments. All authors contributed to the article and approved the submitted version.

## Funding

The authors are grateful for the funding of the National Development and Innovation Office (NKFIH FK 134403 and TKP2021-EGA-42 to ZF) and the support of the Hungarian Brain Research Program (NAP2022-I-8/2022 to ZF). Project no. 2020-2.1.1-ED-2022-00208 has been implemented with the support provided by the Ministry of Innovation and Technology of Hungary from the National Research, Development and Innovation Fund, financed under the 2020-2.1.1-ED funding scheme.

## Conflict of interest

BR was employed by Femtonics Ltd.

The remaining authors declare that the research was conducted in the absence of any commercial or financial relationships that could be construed as a potential conflict of interest.

## Publisher’s note

All claims expressed in this article are solely those of the authors and do not necessarily represent those of their affiliated organizations, or those of the publisher, the editors and the reviewers. Any product that may be evaluated in this article, or claim that may be made by its manufacturer, is not guaranteed or endorsed by the publisher.
