# Supplementary material for: Immunohistological responses in mice implanted with Parylene HT – ITO ECoG devices
Source: Front Neurosci. 2023 Aug 31;17:1209913. doi: 10.3389/fnins.2023.1209913 (PMC10513038; doi:10.3389/fnins.2023.1209913)
Supplement: Supplementary file 1 [file Data_Sheet_1.pdf]

SUPPLEMENTARY

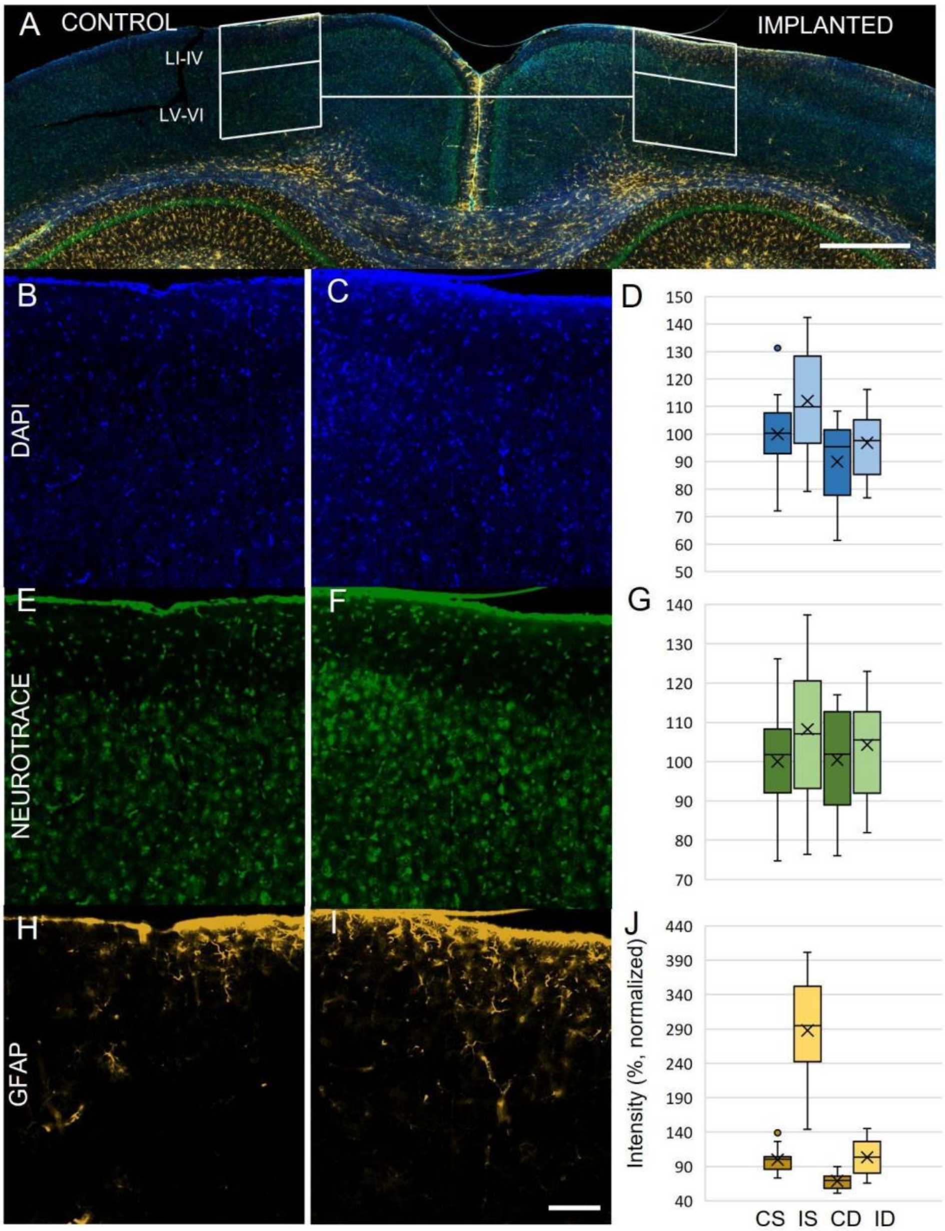

Figure S1: Images of a Parylene HT / ITO ECoG implanted brain after 42 days of chronic implantation (M1). A) Composite image of a coronal slice showing the arrangement of ROIs on control (B, E, H) and implanted (C, F, I) cortex. ROIs are divided into superficial (layers I-IV) and deep (layers V-VI). Magnification 3.2X, scale bar 500 µm. B, C) Cell nuclei stained with DAPI. E, F) Neurons stained with NeuroTrace, a fluorescent Nissl stain. H, I) Astrocytes labeled with GFAP. Magnification 20X, scale bar 50 µm. D, G, J) Comparison of ROI fluorescent intensity of DAPI (D), NeuroTrace (G) and GFAP (J) labeling. Mean ROI intensity was normalized to ROI area and presented here as a percentage of the mean of control, superficial ROIs of their respective stainings. CS - control, superficial. IS - implanted, superficial. CD - control, deep. ID - implanted, deep. Asterisks denote significant differences ($p<0.05$).

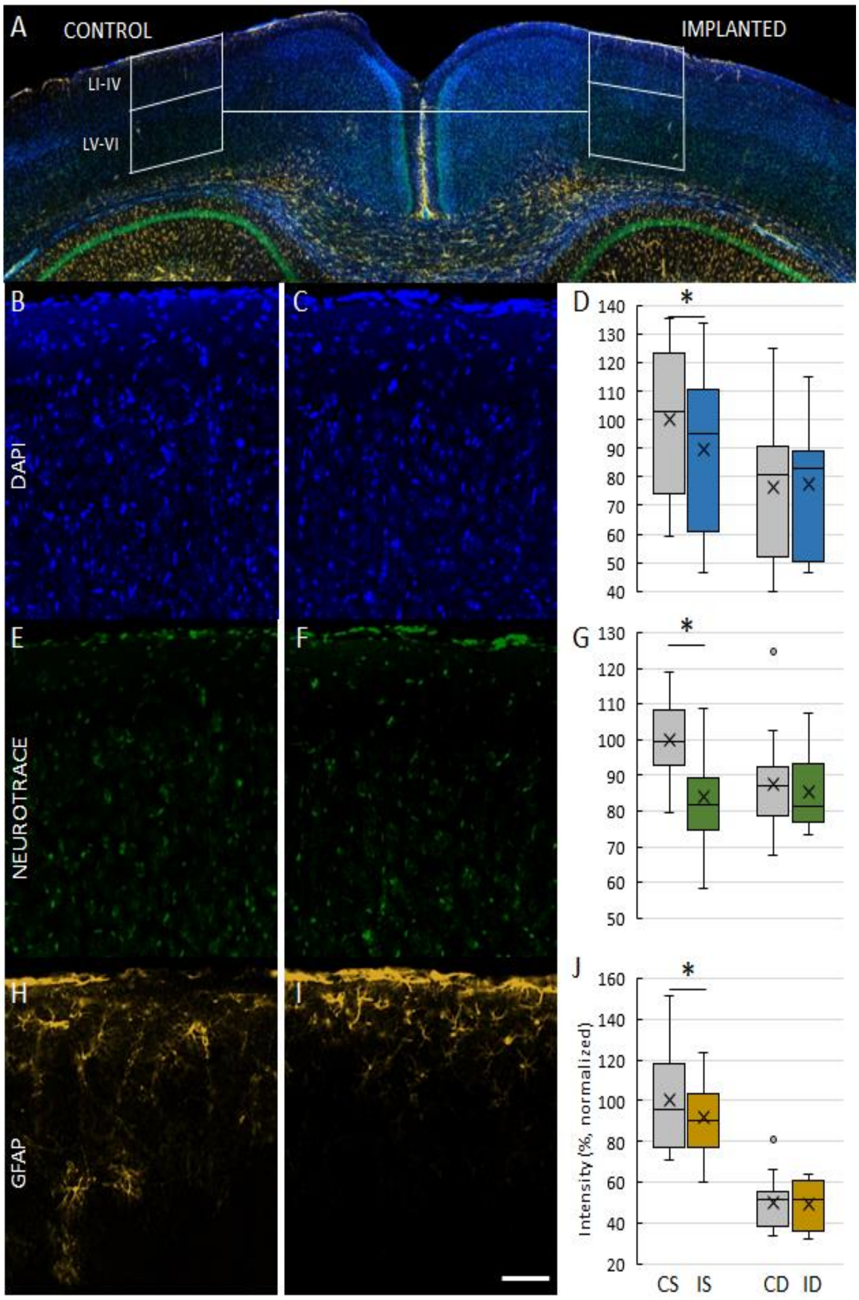

Figure S2: Images of a Parylene HT / ITO ECoG implanted brain after 116 days of chronic implantation (M5). A) Composite image of a coronal slice showing the arrangement of ROIs on control (B, E, H) and implanted (C, F, I) cortex. ROIs are divided into superficial (layers I-IV) and deep (layers V-VI). Magnification 3.2X, scale bar 500 µm. B, C) Cell nuclei stained with DAPI. E, F) Neurons stained with NeuroTrace, a fluorescent Nissl stain. H, I) Astrocytes labeled with GFAP. Magnification 20X, scale bar 50 µm. D, G, J) Comparison of ROI fluorescent intensity of DAPI (D), NeuroTrace (G) and GFAP (J) labeling. Mean ROI intensity was normalized to ROI area and presented here as a percentage of the mean of control, superficial ROIs of their respective stainings. CS - control, superficial. IS - implanted, superficial. CD - control, deep. ID - implanted, deep. Asterisks denote significant differences ($p<0.05$).

Data	Staining	Animal	ROI	Group 1	Group 2	Difference of group means	SD of difference	Statistical test	t - value / W value	Degree of freedom / Number of pairs	p value	Significance level
Fluorescence intensity	DAPI	M1	Superficial	Control	Implanted	12.07 %	18.15 %	Paired t - test	2.772	15	**0.0142**	*
Fluorescence intensity	DAPI	M1	Deep	Control	Implanted	7.58 %	14.26 %	Paired t - test	2.001	15	0.0638	
Fluorescence intensity	DAPI	M2-4	Superficial	Control	Implanted	4.96 %	28.94 %	Wilcoxon matched-pairs signed rank test	530	51	**0.0123**	*
Fluorescence intensity	DAPI	M2-4	Deep	Control	Implanted	3.73 %	28.26 %	Paired t - test	1.577	50	0.1211	
Fluorescence intensity	DAPI	M5	Superficial	Control	Implanted	-10.53 %	26.44 %	Paired t - test	2.985	17	**0.0083**	**
Fluorescence intensity	DAPI	M5	Deep	Control	Implanted	1.44 %	28.20 %	Paired t - test	0.3509	17	0.73	
Fluorescence intensity	NeuroTrace	M1	Superficial	Control	Implanted	8.27 %	17.63 %	Paired t - test	2.151	15	**0.0482**	*
Fluorescence intensity	NeuroTrace	M1	Deep	Control	Implanted	3.79 %	12.78 %	Paired t - test	1.167	15	0.2615	
Fluorescence intensity	NeuroTrace	M2-4	Superficial	Control	Implanted	5.41 %	24.73 %	Paired t - test	2.48	50	**0.0165**	*
Fluorescence intensity	NeuroTrace	M2-4	Deep	Control	Implanted	2.48 %	20.71 %	Paired t - test	1.122	50	0.2671	
Fluorescence intensity	NeuroTrace	M5	Superficial	Control	Implanted	-16.20 %	13.55 %	Paired t - test	4.556	17	**0.0003**	***
Fluorescence intensity	NeuroTrace	M5	Deep	Control	Implanted	-2.50 %	12.67 %	Paired t - test	0.671	17	0.5113	
Fluorescence intensity	GFAP	M1	Superficial	Control	Implanted	187.78 %	71.61 %	Paired t - test	11.75	15	**<0.0001**	****
Fluorescence intensity	GFAP	M1	Deep	Control	Implanted	50.18 %	38.45 %	Paired t - test	5.621	15	**0.0361**	*
Fluorescence intensity	GFAP	M2-4	Superficial	Control	Implanted	11.21 %	38.51 %	Wilcoxon matched-pairs signed rank test	654	51	**0.0018**	**
Fluorescence intensity	GFAP	M2-4	Deep	Control	Implanted	7.56 %	33.94 %	Paired t - test	3.002	50	**0.0042**	**
Fluorescence intensity	GFAP	M5	Superficial	Control	Implanted	-8.13 %	17.74 %	Paired t - test	2.276	17	**0.0361**	*
Fluorescence intensity	GFAP	M5	Deep	Control	Implanted	-2.13 %	22.80 %	Paired t - test	0.5576	17	0.5844	
Density of neurons	NeuroTrace	M1	Both	Control	Implanted	-1.40 %	2.84 %	Paired t - test	1.604	17	0.1272	
Density of neurons	NeuroTrace	M2-4	Both	Control	Implanted	-0.43 %	3.94 %	Wilcoxon matched-pairs signed rank test	-176	51	0.4153	
Density of neurons	NeuroTrace	M5	Both	Control	Implanted	-2.32 %	4.13 %	Paired t - test	2.705	17	**0.015**	*
Cortical thickness	DAPI	M1	NA	Control	Implanted	-32.49 µm	42.13 µm	Paired t - test	3.272	17	**0.0045**	**
Cortical thickness	DAPI	M2-4	NA	Control	Implanted	-23.36 µm	66.34 µm	Wilcoxon matched-pairs signed rank test	-493	51	**0.0201**	*
Cortical thickness	DAPI	M5	NA	Control	Implanted	31.26 µm	33.21 µm	Paired t - test	3.994	17	**0.0009**	***

Supplementary Figure 3. Data on compared groups and statistical tests. * - p<0.05. ** - p<0.01. *** - p<0.001. *** - p<0.0001.
